# Machine learning as a tool to engineer microstructures: Morphological prediction of tannin-based colloids using Bayesian surrogate models

**DOI:** 10.1557/s43577-021-00183-4

**Published:** 2022-02-28

**Authors:** Soo-Ah Jin, Tero Kämäräinen, Patrick Rinke, Orlando J. Rojas, Milica Todorović

**Affiliations:** 1 Department of Chemical & Biomolecular Engineering, North Carolina State University, Raleigh, NC 27695 USA; 2 Department of Bioproducts and Biosystems, Aalto University, Vuorimiehentie 1, P.O. Box 16300, 00076 Espoo, Aalto, Finland; 3 Department of Applied Physics, Aalto University, P.O. Box 11100, 00076 Aalto, Finland; 4 Bioproducts Institute, Departments of Chemical & Biological Engineering, Chemistry, and Wood Science, 2360 East Mall, The University of British Columbia, Vancouver, BC V6T 1Z3 Canada; 5 Department of Mechanical and Materials Engineering, University of Turku, 20014 Turku, Finland

**Keywords:** Tannic acid, Gaussian process regression, Morphology prediction

## Abstract

**Abstract:**

Oxidized tannic acid (OTA) is a useful biomolecule with a strong tendency to form complexes with metals and proteins. In this study we open the possibility to further the application of OTA when assembled as supramolecular systems, which typically exhibit functions that correlate with shape and associated morphological features. We used machine learning (ML) to selectively engineer OTA into particles encompassing one-dimensional to three-dimensional constructs. We employed Bayesian regression to correlate colloidal suspension conditions (pH and p*K*_a_) with the size and shape of the assembled colloidal particles. Fewer than 20 experiments were found to be sufficient to build surrogate model landscapes of OTA morphology in the experimental design space, which were chemically interpretable and endowed predictive power on data. We produced multiple property landscapes from the experimental data, helping us to infer solutions that would satisfy, simultaneously, multiple design objectives. The balance between data efficiency and the depth of information delivered by ML approaches testify to their potential to engineer particles, opening new prospects in the emerging field of particle morphogenesis, impacting bioactivity, adhesion, interfacial stabilization, and other functions inherent to OTA.

**Impact statement:**

Tannic acid is a versatile bio-derived material employed in coatings, surface modifiers, and emulsion and growth stabilizers, which also imparts mild anti-viral health benefits. Our recent work on the crystallization of oxidized tannic acid (OTA) colloids opens the route toward further valuable applications, but here the functional properties tend to depend strongly on particle morphology. In this study, we eschew trial-and-error morphology exploration of OTA particles in favor of a data-driven approach. We digitalized the experimental observations and input them into a Gaussian process regression algorithm to generate morphology surrogate models. These help us to visualize particle morphology in the design space of material processing conditions, and thus determine how to selectively engineer one-dimensional or three-dimensional particles with targeted functionalities. We extend this approach to visualize other experimental outcomes, including particle yield and particle surface-to-volume ratio, which are useful for the design of products based on OTA particles. Our findings demonstrate the use of data-efficient surrogate models for general materials engineering purposes and facilitate the development of next-generation OTA-based applications.

**Graphic abstract:**

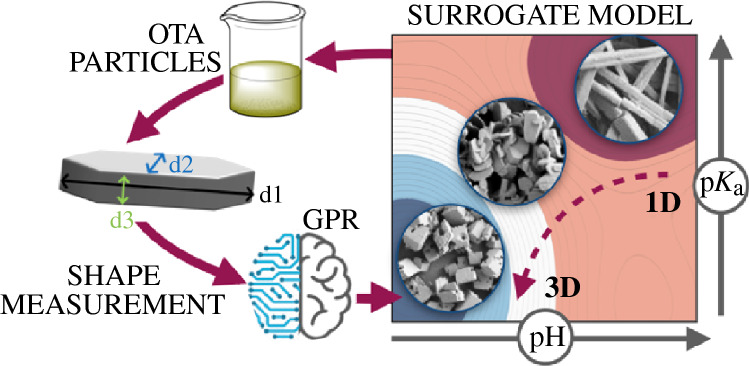

**Supplementary information:**

The online version contains supplementary material available at 10.1557/s43577-021-00183-4.

## Introduction

Tannic acid (TA) is an abundant and versatile bio-based material, which readily affords synthetic pathways for the isolation of its elementary building blocks. TA contains many hydroxyl groups, allowing it to form complexes with different macromolecules via hydrogen-bonding, hydrophobic and cation-π interactions.^1,2^ Abundant hydroxyl groups make TA highly soluble and stable in aqueous solutions. In alkaline conditions, TA undergoes oxidation^3,4^ and produces oxidized tannic acid (OTA) followed by oligomerization. Concomitant oligomerization of OTA leads to the formation of compounds with higher molecular weight and thereby decreases the solubility of the substance.^4^ In this form, OTA can interact with different molecules and serve as coatings,^3,5^ surface modifiers^1,6^ and emulsion stabilizers,^1,3,6,7^ or act as stabilizing and reducing agents to aid in inorganic nanoparticle growth,^8–10^ all the while imparting beneficial biological functionality.^11,12^ For instance, tannic acid has recently been shown to suppress SARS-CoV-2 as a dual inhibitor of the viral main protease.^13^ All these favorable aspects of OTA and other phenolic particles have fueled research into a wide spectrum of applications.^14^

OTA can also be crystallized into particles with structural properties that are highly sensitive to the experimental synthesis. Previously, Bhangu et al.^10^ developed a sonochemical method to chemically transform amorphous tannic acid into nano-/micro-sized crystalline particles without the use of reagents or organic solvents. They obtained OTA particles of different size and shape by simply varying ultrasonic parameters. Kämäräinen et al.^4^ further presented a facile and scalable protocol to prepare OTA of varying morphologies by altering the TA oxidation conditions. The dimensions, shapes, and the yield of these crystalline particles were highly sensitive to initial TA concentration, reaction time, initial pH, and p*K*_a_ of the base.

While OTA particulate constructs can facilitate a range of new applications, particle morphology is a key consideration. In many high surface area systems that incorporate particulate matter, particle morphology and size are major contributors to their overall performance through, for example, relationships between morphology and packing,^15^ percolation,^16^ rheology,^17^ and bioactivity.^18^ Consequently, morphology plays an important role in many applications ranging from heterogeneous catalysts^19^ and electrochemical cells^20,21^ to drug delivery systems.^22^

**Figure 1:**
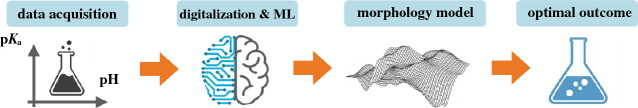
Workflow for machine learning (ML)-guided morphology control of synthesized oxidized tannic acid particles.

In this work, we employ machine learning (ML) to explore the morphology landscape of OTA particles in the chemical design space of processing conditions. As illustrated in **Figure** **1**, we start with OTA synthesis experiments and digitalize them into data points for particle morphology. We apply Gaussian process regression (GPR),^23^ an ML tool for supervised learning, to compute a surrogate model for OTA morphology. Based on the morphology model in the design space of material fabrication, we consider which particle shapes are available and learn how to tune the processing conditions to achieve an optimal outcome for a targeted application.

GPR has been employed in materials science for experimental materials design,^24–30^ often in combination with Bayesian optimization.^31–33^ Given data within the phase space of *N* design parameters, GPR produces the statistically most likely *N*-dimensional landscape, which serves as a surrogate model of a target property.^23^ Gaussian processes (GPs) are capable of good data interpolation, allowing us to build good quality surrogate models with relatively few data points. They produce smooth and continuous landscapes that reflect the continuous chemical process underpinning the data, and can account for experimental uncertainties as data noise. All of these characteristics makes GPR well suited to experimental applications.

The previous study of OTA particle fabrication employed principal component analysis^34,35^ (PCA, an ML tool for unsupervised learning) on experimental data to ascertain that pH and p*K*_a_ used in the OTA solution correlate most strongly with particle shape. We proceed to consider OTA morphology in the two-dimensional (2D) search space of pH and p*K*_a_. Sample characterization was performed by scanning electron microscopy (SEM) imaging. To digitalize the particle shape information, we quantified the physical dimensions allowed by OTA simple crystalline habits and took note of experimental uncertainties.

While PCA is a versatile tool, it was unable to offer further insight into morphology types, nor indicate optimal processing conditions. Conversely, GPR allowed us to visualize OTA particle morphology as a function of pH and p*K*_a_ and delivered a chemically interpretable model. Based on the morphology landscape, our objective was to drive the morphology of particles from one-dimensional (1D) to three-dimensional (3D) shapes. Moreover, by extracting particle yield and volume from each experiment we were able to generate surrogate models for multiple experimental properties at no further cost, allowing us to pursue multi-target tuning of OTA particulate structures. In this article, we present the entire workflow necessary to carry out supervised ML applications on experimental data, with the aim to motivate similar work in the community. Data-efficient ML tools from computer science have the potential to renew experimental practices in materials engineering and boost the search for advanced sustainable compounds.

## Materials and methods

### OTA particle synthesis

**Figure 2:**
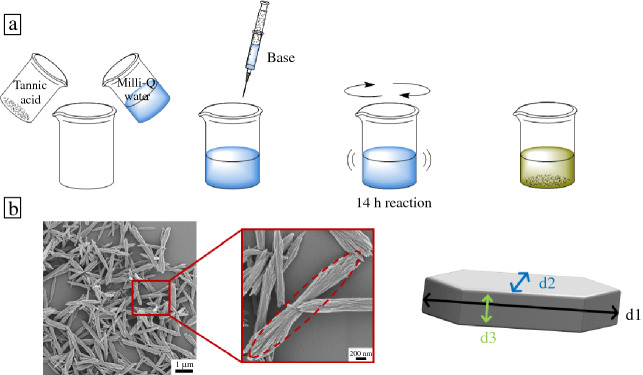
Schematic illustration of the experimental protocol used for data acquisition: (a) oxidized tannic acid colloidal particle synthesis; (b) scanning electron microscope image analysis and particle dimension according to characteristic lengths d_1_, d_2_, and d_3_.

OTA particles were synthesized using the protocol reported previously.^4^ Briefly, aqueous tannic acid solution (2% w/v) was prepared by adding tannic acid powder (1701.20 g/mol, Sigma-Aldrich) into Milli-Q water and rigorously stirring (magnetic bar) until completely dissolved. The pH of the solution was adjusted to a desired pH value with either 1 M KOH, 45% (CH_3_)_3_ N, 1 M NaOH, 0.5 M Na_3_PO_4_ or 25% NH_4_OH (see **Figure** **2**a). Alkaline conditions required for the OTA synthesis reaction to proceed made us select pH > 7, but the base choice was varied widely and resulted in a p*K*_a_ in the range [9.25, 14.9]. All chemicals were reagent-grade and purchased from Sigma-Aldrich. Solutions were covered with perforated Parafilm and were shaken continuously with an orbital shaker for 14 h. All reactions were carried out at room temperature. The grown and precipitated OTA particles were collected and stored at room temperature for further characterization. Despite the simplicity in particle fabrication, multiple experiments were needed to accurately define the conditions that resulted in the given morphology. This required arduous experimentation, as well as time, since each setup produced a specific morphology depending on the reaction conditions.

### SEM image analysis

The synthesized OTA particles were imaged using a field-emission SEM (Sigma VP, Zeiss, Germany) with Schottky emitter at 1.5 kV without stage bias. For this purpose, aqueous suspensions of the OTA particles were cast onto pre-cleaned silicon wafers, dried in ambient laboratory conditions and sputter-coated with 4 nm Pd/Au. All imaging was performed on the same day with the OTA suspensions freshly prepared. Collected SEM images were then analyzed using ImageJ software^36^ to measure the dimensions of the particles typically numbering in the tens (Figure 2b). We measured the length, width and height of OTA particles as d_1_, d_2_, and d_3_, such that d_1_ > d_2_ > d_3_. The average values are reported here as the best estimate of particle dimensions. Standard deviations were recorded to estimate the experimental uncertainty on particle dimensions. All data points, error analysis, and the SEM images are presented in the Supplementary Material document.

### Gaussian process regression algorithm

GPR is a kernel-based algorithm for supervised regression that relies on GP models to represent black box functions.^23^ Given data and the GP prior, Bayes’ rule is applied to compute the GP posterior. The GP posterior mean serves as the surrogate model, the statistically most likely form of the unknown function. The GP posterior variance supplies a local measure of confidence in the model, typically rising in regions of search space where data are scarce and decreasing in well-explored regions.

For GPR fitting we used an uninformative zero mean GP prior and the radial basis function (RBF) kernel to obtain smooth and continuous landscapes. Data noise was Gaussian-distributed with zero mean. To make the model more robust, we applied inverse gamma priors on the hyperparameters of the kernel, the length scale and variance. During regression, the two hyperparameters were fitted in an automated way by maximizing marginal likelihood: this standard GPR procedure ensures that the results do not depend on manual hyperparameter choices.^23^

To compute the surrogate model, we carried out GPR implemented in the Bayesian Optimization Structure Search (BOSS) code. BOSS is an open-source Python code^37,38^ for performing GPR and Bayesian optimization (BO) tasks to solve problems in materials science.^39–42^ It can read pre-recorded data sets or acquire data on-the-fly with acquisition functions. BOSS post-processing capabilities allowed us to construct surrogate model landscapes and analyze their features.

## Results and discussion

We employed 10 experimental data points on crystallized OTA particles collected by Kämäräinen et al.^4^ to initialize the GPR model. In a departure from earlier work, the prospect of supervised learning required us to carry out experimental data analytics and consider different experimental outcomes, as well as measurement uncertainties. Supervised learning calls for a clear outcome, or label, so samples with ill-defined morphologies were not included into the ML model. Another key part of data digitalization was the conversion of experimental observations into customized descriptors for OTA particle morphology.

We started by analyzing the OTA particle morphology landscapes obtained in the 2D search space of pH and p*K*_a_ for shape predictions. To test the predictive power of the model, we performed seven more experiments in key regions of the design space. The additional data also served to refine the morphology model. We validated the morphology landscapes against all experimental data collected, including the samples which were not employed in building the model. Finally, we demonstrated how additional property models for particle yield and volume were built from the same set of experiments and consider multi-objective materials design.

## Experimental data set

The experimental data set was adapted for GPR supervised learning by presenting each point in [***x, y***] pair format. Here ***x*** is the location in the design space of OTA particle processing conditions, and *y* is the label, the morphology design objective for which we construct the surrogate model. Depending on the number of design parameters, ***x*** can be *N*-dimensional. In this work, ***x*** = (*x*_*1*_, *x*_*2*_) with *x*_*1*_ assigned the pH of the solution and *x*_*2*_ the value of base strength p*K*_a_. We limited the design space of the processing conditions (pH, p*K*_a_) to the range of ([7.0, 12.2], [9.0, 15.5]) to reflect the range of the processing conditions within which the experiments were realized.

The morphology of particles was quantified from their measured dimensions (d_1_, d_2_, d_3_). To facilitate comparison between data points, the particle dimension data was scaled by the magnitude of the leading dimension (normalizing the longest dimension to 1.0 for each data point). We defined the morphology label *y* as:

1 $$ \begin{gathered} y = \frac{{d_{2} }}{{d_{1} }} + \frac{{d_{3} }}{{d_{1} }}; \hfill \\ d_{1} = 1.0 \to y = d_{2} + d_{3} \hfill .\\ \end{gathered} $$

This label allows us to distinguish between 1D and 3D morphology conditions as follows:

2 $$ y = \left\{ {\begin{array}{*{20}c} { 0, d_{1} \gg d_{2} , d_{3} ; 1{\rm D}} \\ { 1, d_{3} \ll d_{1} , d_{2} ; 2{\rm D}} \\ { 2, d_{1} \cong d_{2} \cong d_{3} ; 3{\rm D}}. \\ \end{array} } \right. $$

While the 1D–3D signal difference across the realistic particles may be considerably lower than the ideal [0, 2] range, the choice of a physically meaningful property as label *y* allowed us to formulate interpretable surrogate models and gain immediate insight from GPR applications.

**Figure 3:**
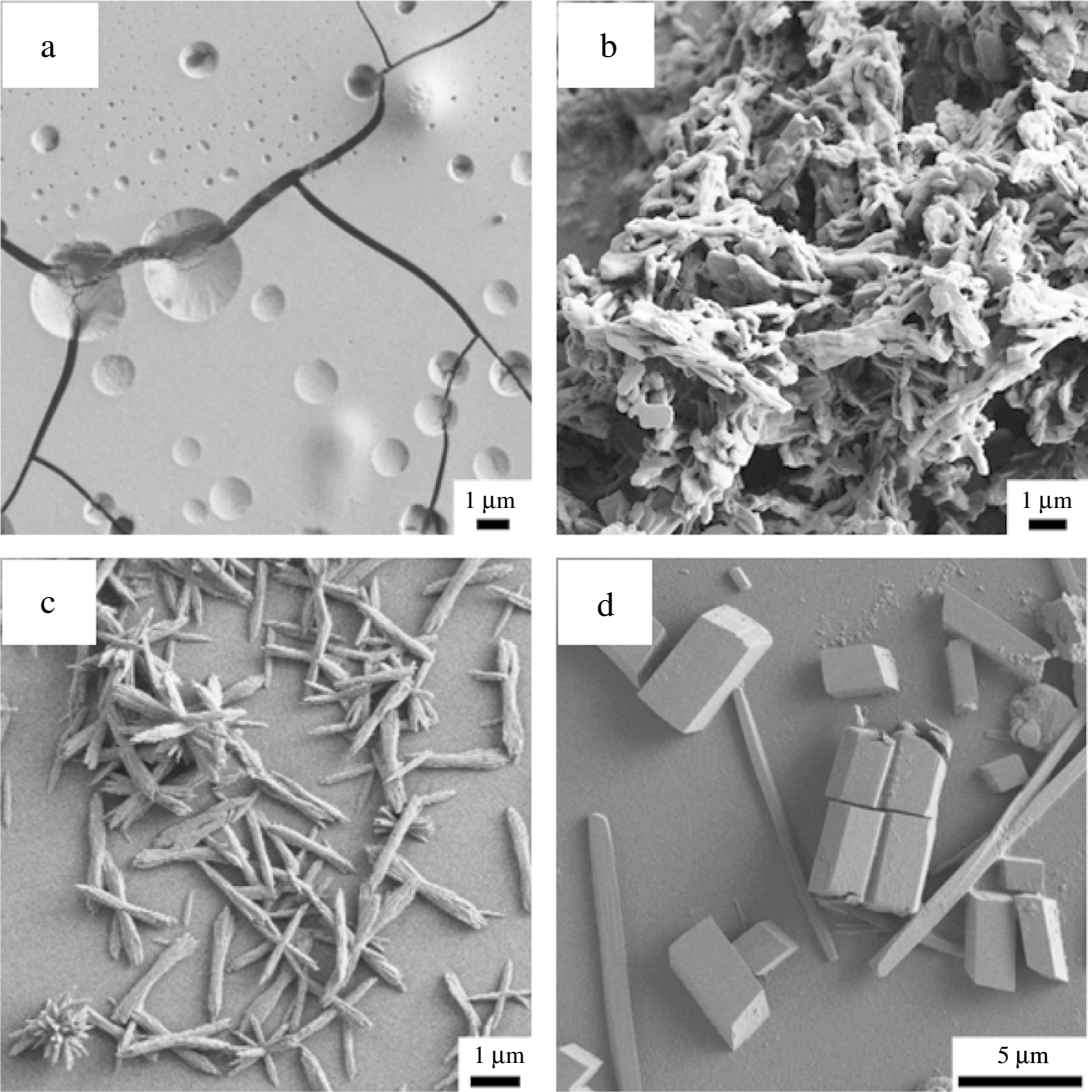
Scanning electron microscope images of precipitated oxidized tannic acid particles: (a) no precipitate; (b) ill-defined morphologies; (c) regular morphology, suitable for characterization; (d) dual morphology.

Next, we review the range of experimental outcomes and discuss their suitability as input for ML application. Unlike in computational research where a numerical result is guaranteed, any experimental data point may result in one of the following outcomes of experimental fabrication, illustrated in **Figure** **3**a: (a) no particle precipitate; (b) non-quantifiable, ill-defined particle morphology; (c) good quality precipitates with quantifiable dimensions; and (d) multi-morphology precipitates. Too many experimental observations in the first two categories would suggest that the chosen design variables are not the key drivers of the material synthesis, and that the experimental design space needs further consideration.

In our work, 74% of experiments (17 points) resulted in quantifiable sample morphology. A further 22% (5) data points featuring ill-defined particle morphology could not be employed in building the model, but served to verify the model predictions. In one case, we observed OTA samples that featured two distinct particle morphologies in comparable yields (Figure 3d). Such a case indicates a saddle-point in the chemical design space, a two-phase region where both morphologies are in coexistence, and should be approached with caution. Here, we characterized the two morphologies and computed their arithmetic average label *y*: such treatment reflected the dichotomy in the design space and was supplying this information in the model.

Experimental uncertainties are common in any practical work, and must be carefully considered. In our study, there were uncertainties associated with both OTA sample fabrication and characterization. While we made every effort to fix all aspects of OTA particle synthesis apart from pH and p*K*_a_, unaccounted differences in ambient conditions such as relative humidity could influence the evaporation rate during the experiments, affecting particle yields and morphologies. Changes in impurity content could also affect the observed morphologies. OTA particle dimensions were measured based on visual assignment of particle boundaries: these may introduce minor uncertainties into the mapping from design space to experimental outcome that are difficult to quantify. Irregular particle sizes in our experiments allowed us to perform a statistical analysis of particle dimensions (and thus morphologies). The standard deviations per particle dimension were combined to compute the overall uncertainty ∆ on the morphology label *y*. Since this quantity reflects the knowability of data, it was adopted to represent all sources of experimental error and served as data noise in the GPR surrogate model (see Supplementary Material for full details). For the precipitate yield, a conservative estimate of 5% variation was assumed.

## Morphology landscapes in the design space

**Figure 4:**
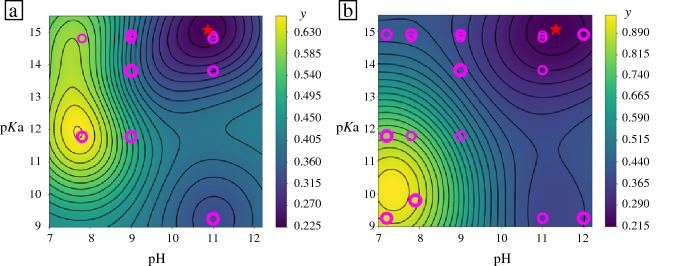
Gaussian process regression surrogate models for morphology label *y* in pH-p*K*_a_ design space fitted with (a) 10 and (b) 17 experimental data points. Chart color reflects the value of the morphology label *y*, with yellow color denoting 3D and dark blue reflecting 1D particle outcome. Magenta circles indicate the loci of the actual experimental data. The red star indicates the processing conditions that produced oxidized tannic acid particles with the most pronounced 1D character (minimum *y* value in the surrogate model).

Based on GPR, we computed the initial surrogate model for OTA particle morphology in the 2D pH-p*K*_a_ design space shown in **Figure** **4**a. The continuous morphology landscape features areas of interest associated with low *y* signal (1D) and high *y* signal (3D) structures. It also indicates that there are regions of design space where no data have been collected and where the model may be less reliable.

The minimum of the surrogate model in Figure 4a suggests that high-pH combined with high-p*K*_a_ produced OTA particles with the most strongly pronounced 1D character ($d_{1} \gg d_{2} , d_{3}$). Conversely, low pH solutions most likely produced 3D particles. To verify these predictions, we sampled further data points at the edges of the design space at pH < 7.8 and pH > 11, and also at low p*K*_a_ values, where data had been sparse. The GPR model that was re-trained with 7 additional experimental points is presented in Figure 4b.

The refined surrogate model for OTA particle morphology retains many of the features of the previous GPR fit in Figure 3a. The predicted high-pH and high-p*K*_a_ conditions for 1D particles remain unchanged. However, the region specific to 3D structures (high *y* values) is now enhanced, shifting to lower p*K*_a_ values. The refined landscape suggests that only low-pH and low-p*K*_a_ processing conditions give rise to 3D particles. The relatively low value of the morphology signal *y* throughout the design space indicates that many experimental outcomes are 1D-like. Particles that are 2D-like may form only in the region of chemical space that neighbors the 3D structural conditions.

## Model validation and predictive power

**Figure 5:**
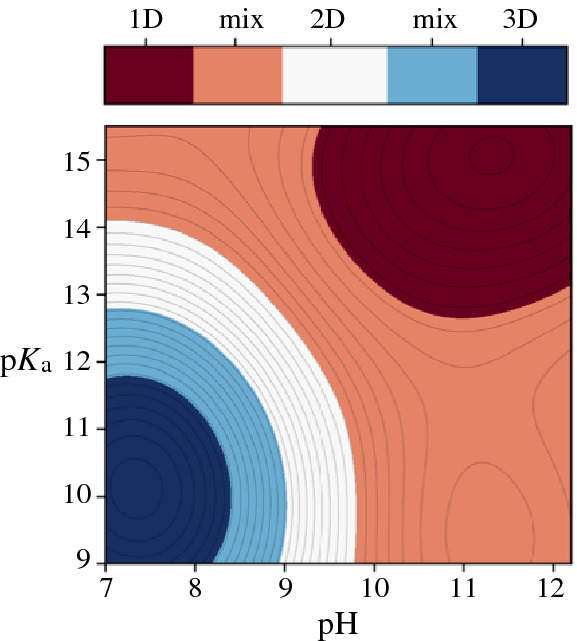
Oxidized tannic acid particle morphology prediction by particle dimensionality, indicating mixed 1D–2D/2D–3D regions.

To extract predictions from the surrogate model, we coarse-grained the landscape into several categories assuming linear progression from 1D to 3D. As illustrated in **Figure** **5**, this allows us to define regions of design space where experiments would reliably produce 1D, 2D, and 3D OTA particles. We observe that 1D and 3D regions of design space are clear and well separated. The model predicts that solution pH and p*K*_a_ are directly correlated: 1D particles are obtained when their values are both high, and 3D when they are both low. In contrast, the 2D particle region spans a limited non-convex area in design space that conforms to the 3D particle region. This implies that 2D particles are difficult to synthesize. The greatest portion of design space was associated with 1D-type structures. The resulting model prediction is that when pH and p*K*_a_ are inversely correlated, 1D-like or 1D-2D mixed morphology particles are expected to occur.

**Figure 6:**
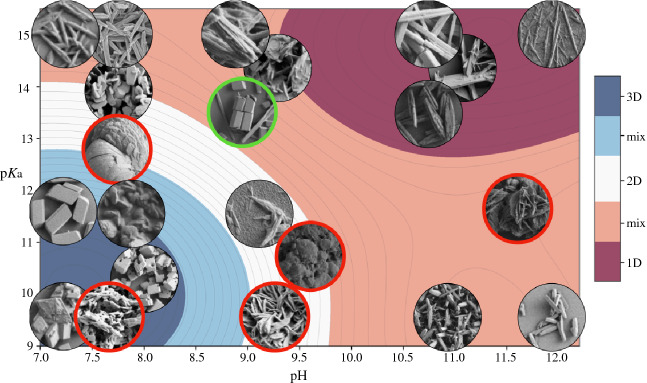
Surrogate model of oxidized tannic acid morphology validated against experimental scanning electron microscope images. Images with red borders indicate experiments with ill-defined morphology while those in green indicate mixed morphologies.

In the next step, we validate our model predictions by cross-referencing SEM images of OTA particles with the particle morphology landscape. **Figure** **6** portrays the landscape overlaid with SEM image data from the area of design space where the OTA particle synthesis was carried out. Images outlined in red represent cases of non-quantifiable particle dimensions (ill-defined morphology), which were not included in the model construction. The case of dual particle morphologies is indicated in green.

It is immediately clear that the predictions regarding 1D and 3D particle formation were correct. 1D landscape regions are associated with very long needle-like particles (up to 0.1 mm), where the design condition $d_{1} \gg d_{2} , d_{3}$ is best satisfied. 1D-like regions exhibit a different 1D morphology where the particles are short and matchstick-like. In some cases, the short 1D particles agglomerate into a larger mass where the morphology is not easily identified. These data were not included into the surrogate model, and yet they correlate well with the mixed morphology 1–2D and 2–3D regions of the landscape. The same is true of the dual morphology data points, which correctly occur in the mixed 1D–2D section of the landscape.

SEM images reveal few examples of 3D particles obtained in these experiments, about 25% of the total. Even fewer are the 2D particle cases, which present mostly as domino-like platelet structures. As predicted by the surrogate model, 1D particles dominate the design space: short matchstick-like structures are the most common experimental outcome. At intermediate pH and p*K*_a_ values, there is a risk of particle aggregation: matchsticks combining into disordered bundles and coral-like growth are observed.

## OTA particle yield and functional properties

Having demonstrated that GPR surrogate models for OTA particle morphology have good predictive power, we turn our attention to other experimental information. With each data point, we recorded the yield of the dried OTA colloidal content. The measurement of particle dimensions further allowed us to analyze and engineer other functional properties such as particle size, volume or surface area. The leading particle length in experiments varied in the range 0.4–130 µm, suggesting that experimental conditions can be used to tailor the particle size to diverse applications. We focused on the ratio of particle surface area to its volume: surface-based chemical processes underpin many technological applications, so maximizing surface area per volume (A/V) complements particle morphology control as an important design objective.

**Figure 7:**
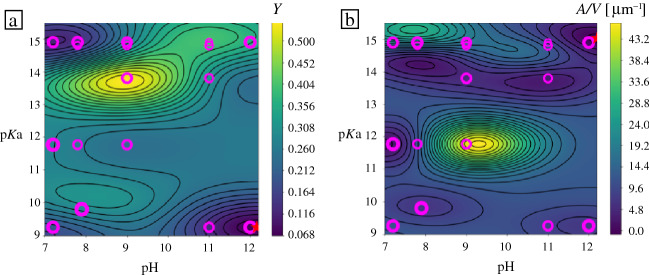
Surrogate models for oxidized tannic acid particle experimental properties: (a) particle yield and (b) particle A/V ratio, in the design space of pH and p*K*_a_ processing conditions. Magenta circles indicate the locations of the experimental data points.

The GPR surrogate model for OTA particle yield is presented in **Figure** **7**a. The irregular features in this landscape suggest that particle yield is strongly correlated with the base employed in the solution, rather than the p*K*_a_ value. For example, applying LiOH (p*K*_a_ 13.8) to OTA leads to relatively high yields, about 60%, but NaOH (p*K*_a_ 14.8) causes the yield to drop below 10 percent. This observation suggests that particle yield may be better correlated with a different property of the base, such as its size. Solution pH does play a role in the particle yield, with largest yields observed in the pH range of 8–11.

The OTA particle A/V landscape, illustrated in Figure 7b, presents a central region where the A/V ratio is very high. These mid-range pH and p*K*_a_ conditions are associated with 2D particles, where experimental data are scarce. OTA particles synthesized in these conditions tend to produce 2D-like lamellar forms that agglomerate into 3D structures (see Figure 6 for SEM images). It was difficult to measure the shape of these particles, so they were not included in the surrogate model. Nevertheless, such samples clearly had the highest A/V ratio, and this was correctly predicted by the A/V surrogate model despite the paucity of data.

Extracting several surrogate models from the same experimental data (at no additional cost) allows us to cross-reference different properties and infer the conditions that would satisfy several design objectives at once. For example, a high yield of 3D particles can be obtained with NH_4_OH in low pH = 7 conditions. Highest yield of 1D OTA particles can be achieved with KOH at pH = 10–11, which also produces largest particles with most surface area exposed. 1D particles with high A/V ratio could be produced at very high p*K*_a_, but at relatively low yields. In further work, different label variables can be arithmetically combined into composite labels and landscapes.

## Discussion and outlook

The purpose of this work was to evaluate the predictive power of GPR on a small experimental data set; therefore, we deliberately constrained the dimensionality of the problem, which also produced interpretable surrogate models. OTA particle morphology is certainly affected by other experimental parameters. Nevertheless, the good predictive power of surrogate models in the relatively simple 2D design space demonstrated that pH and pK_*a*_ alone are sufficient to control particle morphology, in agreement with the earlier PCA result. Unfortunately, PCA was unable to provide insights into the morphology variation that could be achieved with surrogate models.

The morphology landscape portrays a very clear trend, but we are unable to interpret it using scientific intuition. The bottom-up OTA particle synthesis is a result of complex self-assembly where OTA particles coordinate into secondary supramolecular structures, which form tertiary nanofilaments and these assemble into quaternary mesoscopic crystals. It is very difficult to develop any inkling about the outcome of such an intricate procedure, nor about how processing conditions might affect it. Instead, the data-driven landscape can guide further research into the chemical processes behind such outcomes and advance fundamental understanding.

Surrogate models are of general value in materials design because they span all design space, are chemically intuitive and interpretable. It is difficult to establish the criteria for quantitative accuracy of surrogate models. Our work shows that qualitative accuracy already translates to good predictive power, marked by the good visual agreement between the morphology landscapes and the SEM images. OTA samples with ill-defined morphology (not included in the GPR) were particularly important in validating the model predictions. The correspondence of these mixed morphology samples with the appropriate regions on the map demonstrates that good quality ML predictions can be achieved in areas where no experiments were previously performed or included in the model.

The sensitivity of OTA particles to their processing conditions made them an ideal test case for this study, but they remain a challenging material to work with. The composition as well as the molecular structure of tannins are dependent on the source they were extracted from.^43,44^ In other words, the plant species and their physiological state dictate the polydispersity and molecular weight, giving rise to inevitable heterogeneity, which complicates the processing and characterization of the materials. The relatively high experimental uncertainties translated into data noise that amounted to 10% of the entire GPR model corrugation. Such noise did not impair the predictive power of the models in this study, but in other work experimental errors could lead to distorted models and less optimal fits.

The convergence of GP models is an important concern in experimental work where data set sizes are small. Typically, an iterative convergence procedure is followed. Here, the addition of further seven data points intended to verify model predictions had a small effect on the qualitative features of the model, so we stopped short of additional experiments. We note that good quality fits can be obtained with small data sets in the case of simple landscapes (few extrema) and very limited problem dimensionality (2D), thus avoiding the curse of dimensionality.^45^ The need for additional data can be also evaluated from the values of the GPR posterior variance, which tends to decrease with more data included in the model. We considered the OTA morphology model variance after 10 and 17 experimental points (see **Figure S3**). In this work, the relatively large experimental uncertainties translated into large values of GP variance, which remained unchanged with the addition of more data. This finding indicates that in GPR applications to experimental data, where large noise maintains high variance, GP posterior variance might not be a useful measure of model confidence. However, the variance could be used to guide additional experiments.

In further work, our GPR-based approach could be extended to active learning material design workflows. In BO,^32,33^ GPR variance is exploited by acquisition functions to select the sampling location that would most enhance the data set. Acquisition functions balance data exploration (searching less-visited areas of phase space) with data exploitation (searching near optimum points in phase space) to attain search objectives with relatively few data points. Search objectives can be learning the entire landscape or minimizing and maximizing materials properties across the search space.

By demonstrating that GPR performs well with experimental data related to OTA morphology design, this study opens the route toward BO with experimental data in engineering colloids. Integrating BO into experimental work is challenging,^46–48^ but there are many benefits.^49,50^ With acquisition functions guiding the selection of experiments, good predictive power of machine learning could be achieved with fewer experimental data points, facilitating the study of complex *N*-dimensional design spaces with more design variables. Moreover, BO allows to drive experimental data collection towards materials with preferred functional properties (morphological, mechanical or chemical) within the search space. The ML-guided search can thus replace trial-and-error experimental approach in materials design.

## Conclusions

Supramolecular OTA constructs present a prospect of novel applications for this versatile and bioactive material. Controlling particle morphology will help us purpose the OTA particulates toward certain functions and application areas. This study combined materials engineering with GPR supervised machine learning to correlate the processing conditions of OTA colloidal solution with the morphology of the resulting dry OTA particles. The Bayesian surrogate model landscapes revealed the variation of particle morphology in the design space, illustrating the fabrication conditions needed to achieve different particle shapes. The main finding from the OTA morphology landscape is that severe processing conditions (high pH and p*K*_a_) give rise to extended 1D particles with high surface area per volume ratios. Reducing the severity of the solution produces smaller, compact 3D shapes.

Despite the relatively small data set size and large experimental uncertainty, the data-driven morphology landscape was in good agreement with OTA sample images. It exhibited considerable predictive power on samples that were not originally included in the model, marking the potential for predictive materials design. From the same set of experiments, we built surrogate models for OTA particle shape, yield, and surface-to-volume ratio, and cross-referenced them to demonstrate how multiple design objectives could be satisfied at once.

Mapping processing conditions directly to experimental properties of materials constitutes a practical approach to ML-led materials engineering, free of human bias. Such procedures could not only supplant experimental trial-and-error approaches, but also guide further research into the mechanisms of crystallization and self-assembly in complex materials, opening innovative engineering routes toward new phases of matter.

## Supplementary Information

Below is the link to the electronic supplementary material.

Supplementary file1 (DOCX 1312 kb)

## Data Availability

All raw and processed data employed in this study are presented in the Supplementary Material document.
