# Supplementary material for: Machine learning as a tool to engineer microstructures: Morphological prediction of tannin-based colloids using Bayesian surrogate models
Source: MRS Bull. 2022 Feb 28;47(1):29–37. doi: 10.1557/s43577-021-00183-4 (PMC8884090; doi:10.1557/s43577-021-00183-4)
Supplement: Supplementary file 1 — Supplementary file1 (DOCX 1312 kb) [file 43577_2021_183_MOESM1_ESM.docx]

Supplementary Material

for

Machine learning for microstructure control: Bayesian surrogate models predict the morphology of tannin-based colloids

Soo-Ah Jin,^†^ Tero Kämäräinen,^‡^ Patrick Rinke,^§^ Orlando J. Rojas^‡,^,*^ and Milica Todorović^§,||,^*

†Department of Chemical & Biomolecular Engineering, North Carolina State University, Raleigh, North Carolina 27695, United States.

‡Department of Bioproducts and Biosystems, Aalto University, Vuorimiehentie 1, Espoo, P.O. Box 16300, FI-00076 Aalto, Finland.

§Department of Applied Physics, Aalto University, P.O. Box 11100, FI-00076 Aalto, Finland.

^Bioproducts Institute, Departments of Chemical & Biological Engineering, Chemistry, and Wood Science, 2360 East Mall, The University of British Columbia, Vancouver, BC V6T 1Z3, Canada.

||Department of Mechanical and Materials Engineering, University of Turku, FI-20014 Turku, Finland.

**Experimental dataset**

**Table S1.** Experimental dataset, including processing conditions, measured particle dimensions and measurement uncertainties.

|  |  | Processing conditions | | | |  | Particle dimensions [μm] | | |  | Uncertainty [μm] | | |
| --- | --- | --- | --- | --- | --- | --- | --- | --- | --- | --- | --- | --- | --- |
| id |  | base | p*K*_a_ | pH | yield |  | d1 | d2 | d3 |  | ∆d1 | ∆d2 | ∆d3 |
| 1 |  | KOH | 14.90 | 9.0 | 34 |  | 1.60 | 0.25 | 0.25 |  | 0.50 | 0.05 | 0.05 |
| 2 |  | KOH | 14.90 | 11.0 | 47 |  | 20.00 | 0.43 | 0.43 |  | 9.00 | 0.02 | 0.02 |
| 3 |  | NaOH | 14.80 | 7.8 | 7 |  | 1.00 | 0.60 | 0.20 |  | 0.20 | 0.10 | 0.10 |
| 4 |  | NaOH | 14.80 | 9.0 | 24 |  | 4.00 | 1.80 | 0.14 |  | 2.00 | 0.50 | 0.03 |
| 5 |  | NaOH | 14.80 | 11.0 | 35 |  | 12.00 | 2.10 | 0.20 |  | 3.00 | 0.60 | 0.10 |
| 6 |  | LiOH | 13.80 | 9.0 | 64 |  | 6.50 | 1.05 | 0.80 |  | 2.00 | 0.30 | 0.30 |
| 7 |  | LiOH | 13.80 | 11.0 | 28 |  | 9.00 | 1.90 | 1.40 |  | 3.00 | 0.30 | 0.30 |
| 8 |  | Na_3_PO_4_ | 11.77 | 7.8 | 24 |  | 1.10 | 0.70 | 0.30 |  | 0.40 | 0.30 | 0.10 |
| 9 |  | Na_3_PO_4_ | 11.77 | 9.0 | 24 |  | 0.40 | 0.10 | 0.10 |  | 0.20 | 0.03 | 0.03 |
| 10 |  | NH_4_OH | 9.25 | 11.0 | 14 |  | 4.10 | 0.50 | 0.50 |  | 0.60 | 0.10 | 0.10 |
| 11 |  | KOH | 14.90 | 7.2 | 12 |  | 1.70 | 0.22 | 0.22 |  | 0.60 | 0.05 | 0.05 |
| 12 |  | KOH | 14.90 | 7.8 | 14 |  | 2.10 | 0.20 | 0.20 |  | 0.34 | 0.10 | 0.10 |
| 13 |  | KOH | 14.90 | 12.0 | 37 |  | 130.0 | 8.00 | 8.00 |  | 30.0 | 1.00 | 1.00 |
| 14 |  | NH_4_OH | 9.25 | 7.2 | 15 |  | 1.00 | 0.80 | 0.30 |  | 0.30 | 0.40 | 0.10 |
| 15 |  | NH_4_OH | 9.25 | 12.0 | 0.4 |  | 3.00 | 0.60 | 0.60 |  | 1.00 | 0.10 | 0.10 |
| 16 |  | Na_3_PO_4_ | 11.77 | 7.2 | 31 |  | 5.00 | 2.40 | 2.40 |  | 1.00 | 0.70 | 0.70 |
| 17 |  | N(CH_3_)_3_ | 9.80 | 7.9 | 32 |  | 1.40 | 0.90 | 0.60 |  | 0.40 | 0.30 | 0.20 |

**Table S2.** Data points for machine learning in the [**x**, y] format.

|  | Process variables | |  | Target objectives | | | |
| --- | --- | --- | --- | --- | --- | --- | --- |
| id | x_1_ | x_2_ |  | y | ∆y |  | yield |
| 1 | 9.0 | 14.90 |  | 0.3125 | 0.0625 |  | 34 |
| 2 | 11.0 | 14.90 |  | 0.0430 | 0.0020 |  | 47 |
| 3 | 7.8 | 14.80 |  | 0.8000 | 0.2000 |  | 7 |
| 4 | 9.0 | 14.80 |  | 0.4850 | 0.1325 |  | 24 |
| 5 | 11.0 | 14.80 |  | 0.1917 | 0.0583 |  | 35 |
| 6 | 9.0 | 13.80 |  | 0.3667 | 0.0667 |  | 64 |
| 7 | 11.0 | 13.80 |  | 0.9091 | 0.3636 |  | 28 |
| 8 | 7.8 | 11.77 |  | 0.5000 | 0.1500 |  | 24 |
| 9 | 9.0 | 11.77 |  | 0.2439 | 0.0488 |  | 24 |
| 10 | 11.0 | 9.25 |  | 0.2846 | 0.0923 |  | 14 |
| 11 | 7.2 | 14.90 |  | 0.2588 | 0.0588 |  | 12 |
| 12 | 7.8 | 14.90 |  | 0.1905 | 0.0952 |  | 14 |
| 13 | 12.0 | 14.90 |  | 0.1231 | 0.0154 |  | 37 |
| 14 | 7.2 | 9.25 |  | 1.1000 | 0.5000 |  | 15 |
| 15 | 12.0 | 9.25 |  | 0.4000 | 0.0667 |  | 0.4 |
| 16 | 7.2 | 11.77 |  | 0.9600 | 0.2800 |  | 31 |
| 17 | 7.9 | 9.80 |  | 1.0714 | 0.3571 |  | 32 |
| max. | 12.00 | 14.90 |  | 1.10 | 0.50 |  | 64.0 |
| min. | 7.20 | 9.25 |  | 0.04 | 0.00 |  | 0.4 |
| range | 4.80 | 5.65 |  | 1.06 | 0.50 |  | 63.6 |
| average | 9.23 | 12.90 |  | 0.48 | 0.15 |  | 26.0 |
| stdev. | 1.74 | 2.32 |  | 0.35 | 0.14 |  | 15.5 |

**SEM image library for quantifiable OTA colloidal particles**


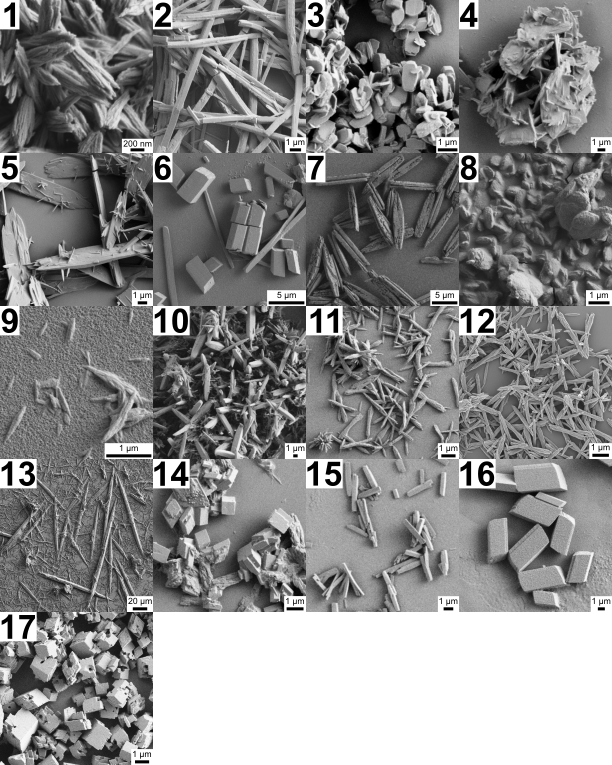


**Figure S1.** SEM micrographs of OTA particle samples with well-defined morphology. Particle dimensions and measurements uncertainties are listed in Tables S1 and S2.

**SEM image library for unquantifiable OTA colloidal particles**

Table S3. Experimental dataset

| id |  | base | p*K*_a_ | pH |  |
| --- | --- | --- | --- | --- | --- |
| U1 |  | LiOH | 13.80 | 7.8 |  |
| U2 |  | Na_3_PO_4_ | 11.77 | 11.0 |  |
| U3 |  | NH_4_OH | 9.25 | 7.8 |  |
| U4 |  | NH_4_OH | 9.25 | 9.0 |  |
| U5 |  | N(CH_3_)­_3_ | 14.80 | 9.0 |  |
| U6 |  | N(CH_3_)­_3_ | 9.80 | 11.0 |  |


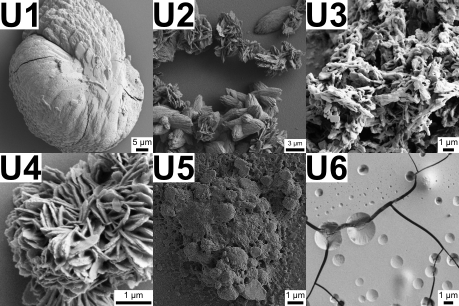


**Figure S2.** SEM micrographs of OTA particle samples with ill-defined morphology or where no precipitate was observed. No particle dimension were recorded for these samples, but the micrographs were employed to validate the GPR model.

**Supplementary information on the GPR surrogate models**


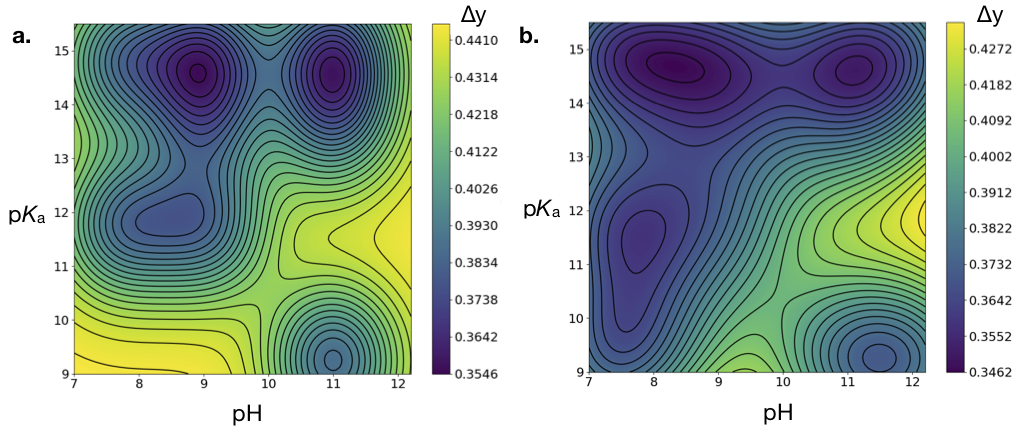


**Figure S3**. GP posterior variance corresponding to GP posterior mean presented in **Figure 4** in the main manuscript.
